# Ligature of the Carotid Artery for Hemiplegia, with a Case in Illustration

**Published:** 1831

**Authors:** 


### XXIII.

LIGATURE OF THE CAROTID ARTERY FOR HEMIPLEGIA, WITH A CASE IN ILLUSTRATION. By MR. PRESTON, Assistant-Surgeon, General Depôt of European Pensioners, Cuddalore.

THE paper which has been transmitted to us under the above title, was too detailed and voluminous for insertion in this journal, and therefore we have given an abridgment of it, reserving the original document for the author, if he claims it.

*Case.* Peter Rachford, ætat. 50, was admitted into the Depôt Hospital, on the 25th October, 1830, with the following symptoms:—loss of power and sensation in the left side of the body—left cheek droops, and is drawn towards the opposite, when he attempts to speak, which is now almost impracticable—pain in the paralytic leg—head free—circulation natural—skin cold—tongue clean—bowels open. The foregoing symptoms came on suddenly the preceding night. The man has always been a hard liver, and had been much intoxicated about a week before the seizure. A blister was applied to the head and ten grains of calomel were exhibited twice a day. On the 28th the mouth was slightly affected; and the calomel being continued, salivation ensued. About this time iodine was also given, and a seton was inserted in the nape of the neck. On the 13th November, there being no mitigation of the paralytic symptoms, we find the nux vomica was exhibited. On the 17th he evinced pain and tenderness in the region of the liver, for which he was leeched and blistered. The pain was removed, but the other symptoms remained nearly the same.

On the 22d November, the paralytic complaint being in statu quo, Mr. Preston determined on tying the carotid artery of the right side, in conformity with the general fact, that the cause of the paralysis is in the opposite side of the head. The steps of the operation need not be detailed. In the evening of the same day, there was no perceptible inconvenience from the operation.

23d. Slept pretty well last night—skin warm—pulse 100, and not intermitting, as it had generally done before—tongue thickly furred, but moist—slight cough, and uneasiness in the chest. There was also some difficulty of deglutition. In the evening, the pulse was down to 80, and soft—skin warm and moist—bowels confined. Ol. ricini statim.

24th. Pulse 60, irregular, and intermitting. Complains of more pain in the paralytic arm.

26th. He appeared to speak more distinctly, and in two days more spoke as distinctly as at any period of his life. On the first of December he was put upon half-diet. 4th Dec. Has considerably recovered the power of the left leg, which he is able to draw up and down. He this day attempted to walk, but did not succeed. The arm is still completely powerless. The appetite is good, and he sleeps well. 12th. December, we find the patient able to walk alone, with the aid of a stick. The arm is still paralytic. 15th. The wound is quite healed, except at the spot where the ligature protrudes. He was discharged the hospital on the 30th of December, at his own request, being able to walk about very comfortably with the assistance of a stick, but the paralyzed arm still without any muscular power.

The following are Mr. Preston's reasons for the operation.

"In tying the carotid artery for palsy, I have deviated entirely from the treatment pursued in that disease, and was led to undertake the operation from the consideration which I subjoin.

I conceived generally that it might be had recourse to, with great advantage, in diseases of the brain, especially such as we had reason to believe, depended upon congestion, inflammation or irritation of that organ; as its principal effect would be, to diminish its supply of blood — an object we have more or less in view in the treatment of cerebral affections; although it cannot always be accomplished by depletory measures.

Venesection and the application of leeches often increase the determination to the head; partly by the disturbance they excite generally in the system; and partly by the reaction which frequently follows their employment.

It appeared to me also that a more durable, and more decided effect would be induced by this operation than by any mode of depletion; and that it might entirely remove, or greatly diminish congestion and chronic inflammation of the brain and its membranes; the causes, I believe of many diseases, which the common modes of treatment too frequently fail of relieving.

As palsy may be induced by some change in the brain itself, either functional or organic, without any complication of the spinal marrow, it appeared to me a disease likely to be affected by this operation; and as, from the suddenness of the attack in the present case, I was inclined to attribute it to some such change,—(I conceived extravasation within the cranium) — it seemed a favourable opportunity for trying its effects. I believed that the pressure caused by the extravasated fluid, might be removed by the diminution in the volume of blood sent to the head; which would be affected by tying one of the carotid arteries. The paralysis too, I was aware might depend upon a very different cause from that conjectured, and yet be removed by this operation: for we know too little of the diseases of the brain and its different affections, to be certain in every case of their precise nature, and to predict always with accuracy the result of the measures we employ with a view of removing them. If the palsy arose from congestion, inflammation or irritation, it appeared equally eligible as in the case supposed by me; but my principal reason for undertaking it in the present instance, supported by the arguments I have already adduced, was, that the case appeared otherwise hopeless; the disease being altogether unaffected by the remedies I had employed and the patient's strength beginning to sink.

Obliteration of the common carotid artery on one side has been frequently effected, without any ill consequences attributable solely to it, and without any disturbance of the brain, heart or lungs. How far it would be safe to tie this artery on both sides, is a question of very serious consideration. It has been done repeatedly on the dog by Bichat, and death occurred in only two instances. I have myself tied both these arteries twice in the same animal but without producing any effect.

Under desperate circumstances, and at an interval of some time after tying the first; we would, provided no relief followed, and the case otherwise hopeless, I conceive, be justified in tying the second. It might, however, be done with a slip knot, or in such a manner as to admit of the ligature being loosened, should any alarming symptoms follow, or the ligature might be applied so as to intercept only a portion of the blood, sent to the head by narrowing, at the part, the calibre of the artery—we might thus easily increase or diminish according to circumstances the column of blood which it transmits.

The operation itself excites no constitutional disturbance, or but very little.

I quote the passage from Bichat—in which however there is some obscurity, probably from some error of the translator—as also the same, worded somewhat differently, and found in Rees's Encyclopædia, article—heart.

From Bichat.—

'It is easy to prove that the movement of the blood is necessary to that of the brain, expose the brain of an animal in part and tie the carotids.

In such case, the cerebral movement will be sometimes weakened, and then the animal will be stupified, at other times the vertebral arteries will exactly supply the place of the carotids, and then there will be nothing deranged in the principal functions of the brain: for there is always a relation existing between the alternate rise and fall of the cerebral mass and the energy of life which it displays.

*In general, the obliteration of the carotids is never suddenly mortal. Animals will live without them, for at least a considerable time. I have kept dogs in this state for several days,* and have afterwards made use of them for other experiments; two however died in the course of six hours after the application of the ligatures.'—*Gould's Translation*, page 157.

The part that seems obscure in the foregoing paragraph I have underlined. The number of dogs that died after this operation is not specified, nor at what interval of time.

The same passage is thus worded in Rees's Encyclopædia.

'The heart, which propels the red blood, affects the brain by the motion which it communicates to that organ. If the arrival of this fluid be completely intercepted the motion of the brain ceases, and life is extinguished; where the carotids alone are tied, the vertebral arteries still keep up the movement and no ill effect is produced.'

The diseases in which I conceive this operation most likely to prove serviceable, are, such as depend upon determination of blood to the brain, congestion, inflammation, or irritation of that organ; but it must be understood, that I propose it as a remedy only after other means have failed. I may here specify apoplexy, phrenitis, hydrocephalus, many cases of injuries of the head, palsy, epilepsy, and insanity; which latter disease is now generally admitted — indeed known to depend upon a change of some kind in the brain or its membranes; most commonly some modification of inflammation or its products. Where this organ in insanity is affected only from sympathy with some other; how far tying one of the carotid arteries would be serviceable must, I fancy, depend upon considerations connected with each particular case. Here, as in more dangerous diseases, both these vessels might be tied with the precautions already stated; allowing a longer interval of time to elapse between the operations. There are some other points connected with this subject upon which I am at present unable to enter, but which upon a future occasion I hope to lay before the Board."

